# Inhibition activity of a disulfide-stabilized diabody against basic fibroblast growth factor in lung cancer

**DOI:** 10.18632/oncotarget.15556

**Published:** 2017-02-21

**Authors:** Yaxiong Cai, Shuange Yao, Jiangchuan Zhong, Jinxia Zhang, Haowu Jiang, Yanrui Deng, Ning Deng

**Affiliations:** ^1^ Guangdong Province Key Laboratory of Molecular Immunology and Antibody Engineering, College of Bioscience and Technology in Jinan University, Guangzhou, China

**Keywords:** ds-diabody, bFGF, lung cancer, angiogenesis, lymphangiogenesis

## Abstract

The over-expression of basic fibroblast growth factor (bFGF) plays a crucial role in the development, invasion and metastasis of lung cancer. Therefore, neutralizing antibodies against bFGF may inhibit the growth of lung cancer. In this study, a Disulfide-stabilized diabody (ds-Diabody) against bFGF was constructed by site-directed mutation and overlap extension PCR (SOE-PCR) at the position of VH44 and VL100 in the scFv. The ds-Diabody was constructed and expressed in *Pichia pastoris*. We found that the ds-Diabody against bFGF could efficiently suppress the proliferation, migration and invasion of human lung cancer A549 cells *in vitro*. Moreover, in A549 cells, the ds-Diabody against bFGF could inhibit bFGF-induced activation of downstream signaling regulators, such as phospho-Akt and phospho-MAPK. In the nude mouse xenograft model of lung cancer, the ds-Diabody against bFGF could significantly inhibit tumor growth and decrease the densities of micro-vessels and lymphatic vessels in tumor tissue. Our data indicate that the ds-Diabody against bFGF could effectively suppress the lung cancer growth through blockade of bFGF signaling pathway and inhibition of tumor angiogenesis, which may make it a potential therapeutic candidate antibody drug for human lung cancer therapy.

## INTRODUCTION

Basic fibroblast growth factor (bFGF) is a pleiotropic growth factor. One of the most important functions of bFGF is to promote endothelial cell proliferation and promote angiogenesis by the physical organization of endothelial cells into tube-like structures [1–3]. bFGF is overexpressed in malignant tumors [4–7]. The binding of bFGF and its receptor (FGFR) may activate the signaling pathways of MAPKs/ERKs and PI3K/AKT, which is correlated with tumor growth, migration, angiogenesis and lymphangiogenesis [4, 5]. Therefore, blocking bFGF/FGFR activity with antibodies might be a good therapeutic strategy for tumor patients [8].

The anti-bFGF murine monoclonal antibodies (mAbs) have been reported to have anti-tumor effect on chondrosarcoma, glioma, Lewis lung carcinoma and melanoma [9–12]. Li proved that traditional anti-bFGF murine mAbs display remarkable anti-tumor effect on B16 melanoma *in vivo* and *in vitro* [12]. However, traditional murine mAbs could induce human anti-mouse antibody (HAMA) response and interfere with the therapeutic effect [13–15]. It is very important to construct human antibodies to avoid HAMA reaction. Tao has prepared a full-length human antibody against bFGF, which could remarkably inhibit the growth of melanoma *in vitro* and *in vivo* [16].

Small molecule antibodies get more and more attention for its good tissue penetration and low immunogenicity. They may have a potential application in target therapy of human diseases [17]. Diabody is one of the small molecule antibodies, which is a non-covalently associated bivalent molecule, created from scFv by shortening the polypeptide linker between the VH and VL domains [18]. The diabody was non-covalently associated and the linker may interfere with the antigen binding, which may result in lower affinity and unstable [19]. Introduction of disulphide bond in the framework of VH and VL domains could stabilize the diabody and keep the affinity [17, 20–22].

In this study, we mainly reported the construction of ds-Diabody and the inhibition effect and the potential mechanisms of the human disulfide-stabilized diabody against bFGF on the growth of human lung cancer A549 cells *in vitro* and *in vivo*.

## RESULTS

### Expression and purification of the ds-Diabody against bFGF

**Figure 1:**
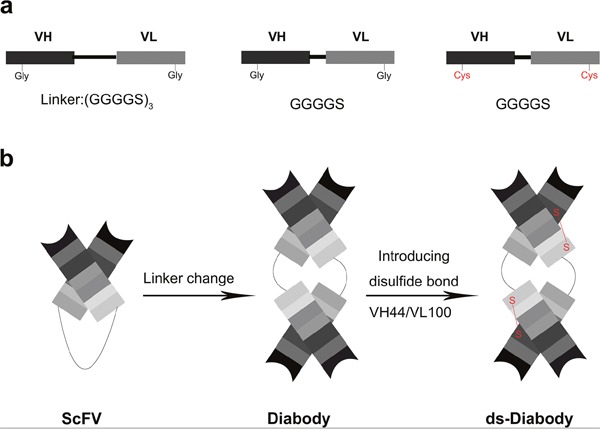
Construction of the ds-Diabody against bFGF **a.** The ds-Diabody against bFGF was constructed by introducing disulfide bonds between VL and VH. **b.** Schematic representation of the construction of ds-Diabody against bFGF

**Figure 2:**
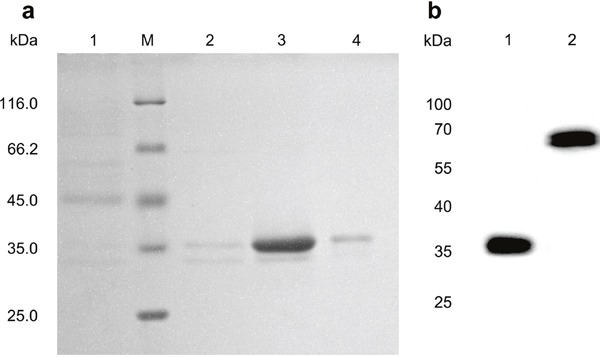
Purification and identification of the ds-Diabody against bFGF by SDS-PAGE and western-blot **a.** SDS-PAGE of ds-Diabody against bFGF. Lane M: Protein molecular weight marker; Lane 1: Proteins from culture supernatant; Lane 2: Other proteins; Lane 3: Fractions obtained by Ni Sepharose affinity chromatography and anion-exchange chromatography; Lane 4: Other proteins. **b.** Western-blot assay of ds-Diabody against bFGF. Lane 1: Western blot assay of the ds-Diabody against bFGF under reducing condition; Lane 2: Western blot assay of the ds-Diabody against bFGF under non-reducing condition

The high affinity human antibodies of scFv against bFGF were selected from a phage display library [16]. The human disulfide-stabilized diabody against bFGF (ds-Diabody) gene fragment was constructed by site-directed mutation and overlap extension (SOE-PCR) at the VH44 and VL100 position of the scFv (Figure 1). The ds-Diabody gene fragment was constructed into the yeast expression vector pPICZαA and the recombinant plasmid pPICZαA-ds-Diabody was transformed into *Pichia pasporis* strain GS115. The ds-Diabody against bFGF could be high level expressed in yeast. The yield of recombinant ds-Diabody against bFGF could reach 30-50 mg/L in cell culture. The result of western-blot showed that the ds-Diabody against bFGF was specific appeared at the molecular weight of approximately 35 kDa under reducing condition and 70 kDa under non-reducing condition (Figure 2b).

The ds-Diabody against bFGF was secretion expressed in the supernatant of recombinant yeast and purified by Ni Sepharose^TM^ 6 affinity chromatography and anion-exchange chromatography. The high purity of recombinant ds-Diabody against bFGF was obtained and the purity of it is above 95% (Figure 2a).

### Antigen binding activity of the ds-Diabody against bFGF

**Figure 3:**
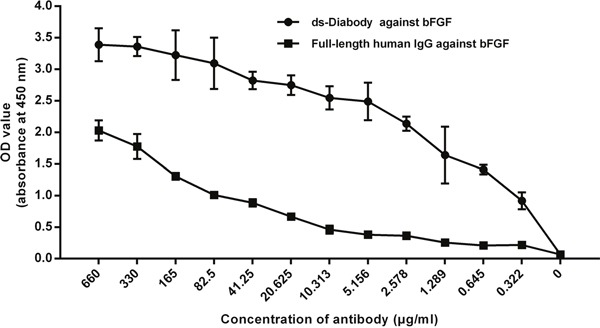
Antigen binding activity of the ds-Diabody and full-length human antibody against bFGF were assayed by indirect ELISA

The antigen binding activity of the ds-Diabody against bFGF was analyzed by indirect ELISA. When the concentration of the antibodies was 0.332 μg/mL, the value of OD_450 nm_ of the ds-Diabody could reached about 1.0, while the value of OD_450 nm_ of the full-length human antibody was just under 0.1. The results showed that the ds-Diabody against bFGF could specifically bind to bFGF and the formation of disulphide bonds in the ds-Diabody did not influence its antigen binding activity (Figure 3).

### Proliferation inhibition of A549 cells by the ds-Diabody against bFGF

**Figure 4:**
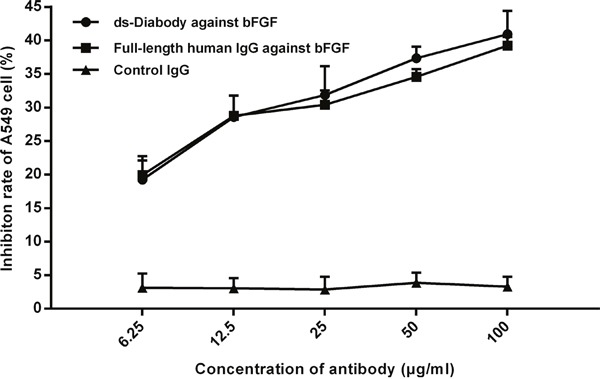
Proliferation inhibition effects of the ds-Diabody against bFGF on lung cancer cells The lung cancer A549 cells (2×10^3^ cells/well) were transferred to 96-well plates and treated with 15 ng/mL bFGF plus ds-Diabody at serially diluted concentrations. The results of CCK-8 showed that ds-Diabody against bFGF could inhibit the proliferation of lung cancer cells in dose dependent. The data were represented as the mean±SD of three independent experiments performed in triplicate

The proliferation inhibition assay of A549 cells was conducted by CCK-8 kit. The results of cell proliferation inhibition assay showed that the cell viability was decreased with the increasing of the ds-Diaboy against bFGF. When the concentration of the ds-Diabody was 6.25, 12.5, 25, 50 and 100 μg/mL, the cell proliferation inhibition rate was about 19.23%, 28.59%, 31.88%, 37.35 % and 40.94% respectively. The results indicated that the ds-Diaboy could inhibit the proliferation of human lung cancer A549 cells in a dose-dependent manner (Figure 4). The positive control of full-length human IgG against bFGF showed similarly inhibitory effect on the proliferation of A549 cells and the irrelevant IgG showed no inhibitory effect (Figure 4).

### Blocking of bFGF-triggered phosphorylation of Akt and MAPK by the ds-Diabody against bFGF

**Figure 5:**
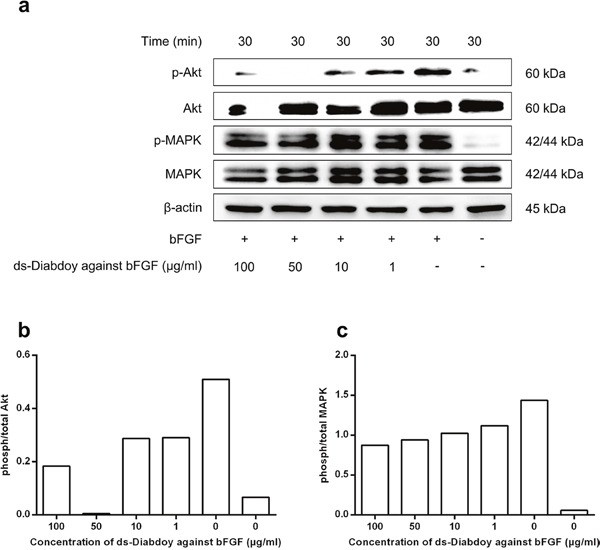
Western-blot assays of Akt and MAPK phosphorylation in lung cancer cells treated with the ds-Diabody against bFGF The A549 cells (2×105 cells/well) transferred in 6-well plates were serum-starved cultured and treated with 15 ng/mL bFGF and serially concentrations of the ds-Diabody and incubated for 30 min. The cell lysates were collected and transferred in PVDF membrane for western-blot assay. The primary antibodies were anti-MAPK, anti-p-MAPK, anti-Akt and anti-p-Akt. The β-actin was served as the reference control. **a.** Akt and MAPK phosphorylation of human lung cancer A549 cells treated by various concentrations of ds-Diabody against bFGF (1-100 μg/mL) for 30 min. **b.** Quantitative analysis of phosphorylated/total Akt. **c.** Quantitative analysis of phosphorylated/total MAPK

The starved A549 cells were treated with different concentrations of ds-Diabody together with bFGF. The cells were lysed and the proteins in the lysates was separated with SDS-PAGE and assayed with western-blot. The western-blot results showed that the phosphorylation of Akt and MAPK could be activated by bFGF in A549 cells, and the ds-Diabody agasint bFGF could effectively block the phosphorylation activation of Akt and MAPK in a dose-dependent manner (Figure 5). The results revealed that the ds-Diabody against bFGF could effectively suppress the proliferation of A549 cells by blocking the signal pathways of Akt and MAPK.

### Migration inhibition of A549 cells by ds-Diabody against bFGF

**Figure 6:**
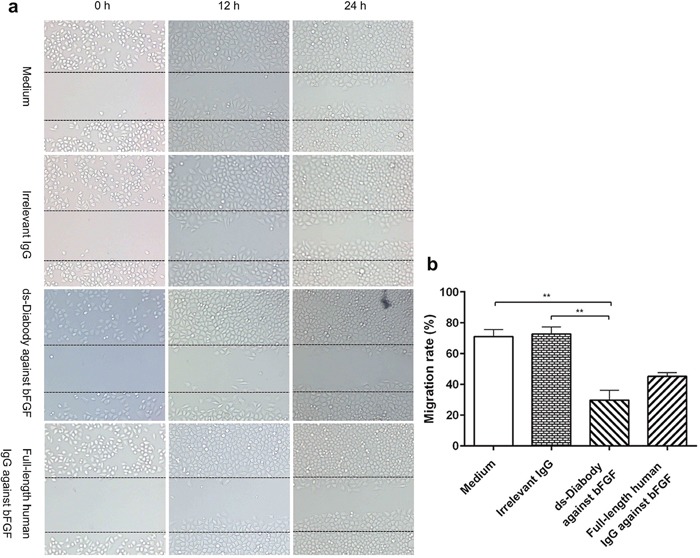
The migration inhibition of A549 cells by ds-Diabody against bFGF **a.** The migration of A549 cells in different conditions at different times. **b.** The quantitative analysis of the migration rate of different groups. The results indicated that the ds-Diabody against bFGF could significantly inhibit the migration of A549 cells when compared with the Irrelevant IgG group (*P < 0.05, **P < 0.01)

The human lung cancer A549 cell was a kind of tumor cell with high migration capability [23]. When subjected to scratches, the A549 cells could migrate quickly to the blank area of scratch and repair the scratches. The migration rate of A549 cells after scratch treatment for 24 h in groups of the ds-Diabody against bFGF, the full-length human IgG against bFGF, the irrelevant IgG and medium alone were 29.72%, 45.24%, 72.59% and 71.03% respectively (Figure 6). The results of scratch assay indicated that the ds-Diabody against bFGF could significantly inhibit the migration of A549 cells *in vitro*.

### Invasion inhibition of A549 cells by the ds-Diabody against bFGF

**Figure 7:**
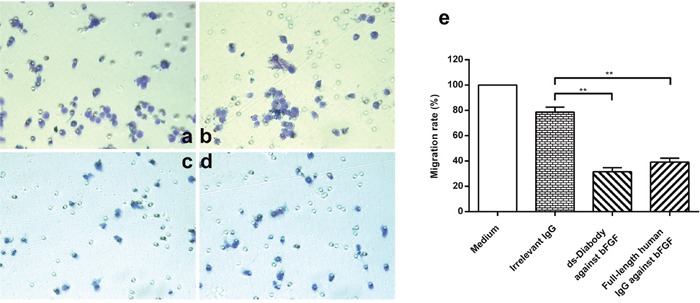
The invasion inhibitory of A549 cells by the ds-Diabody against bFGF The cells were cultured in serum-free medium containing 15 ng/mL bFGF in the upper chamber and were chemo-attracted by the 10% serum in the lower chamber. The invasion cells migrated into the lower side were stained by crystal violet and imaged with a computerized imaging system. **a.** A549 cells were treated with DMEM serum-free medium. **b.** A549 cells were treated with the irrelevant IgG. **c.** A549 cells were treated with the ds-Diabody against bFGF. **d.** A549 cells were treated with the full-length human antibody against bFGF. **e.** The quantitative analysis of A549 invasion. The number of cells observed from serum-free DMEM medium group was set as 100. The data were represented as the means ± SD. *P<0.05; **P<0.01

The invasion assay of A549 cells was conducted in a transwell with matrigel. The A549 cells (5×10^4^ cells/well) were plated into the transwell with a layer of matrigel. The cells were serum-starved cultured to minimize any interference of serum growth factors, and stimulated with 15 ng/mL bFGF and 100 μg/mL ds-Diabody against bFGF for 16 h. In the upper chamber, the cells were cultured with serum-free medium containing 15 ng/mL bFGF and the lower chamber were the medium with 10% serum. The cells may be chemo-attracted by the serum in the lower chamber and result in invasion. The invasion ratio of A549 cells in the group of ds-Diabody against bFGF was about 31.56% while the negative control group was 78.65%. The results indicated that the ds-Diabody against bFGF could significantly inhibit the invasion of A549 cells (Figure 7).

### Tumor growth inhibition of the ds-Diabody against bFGF in mice model

**Figure 8:**
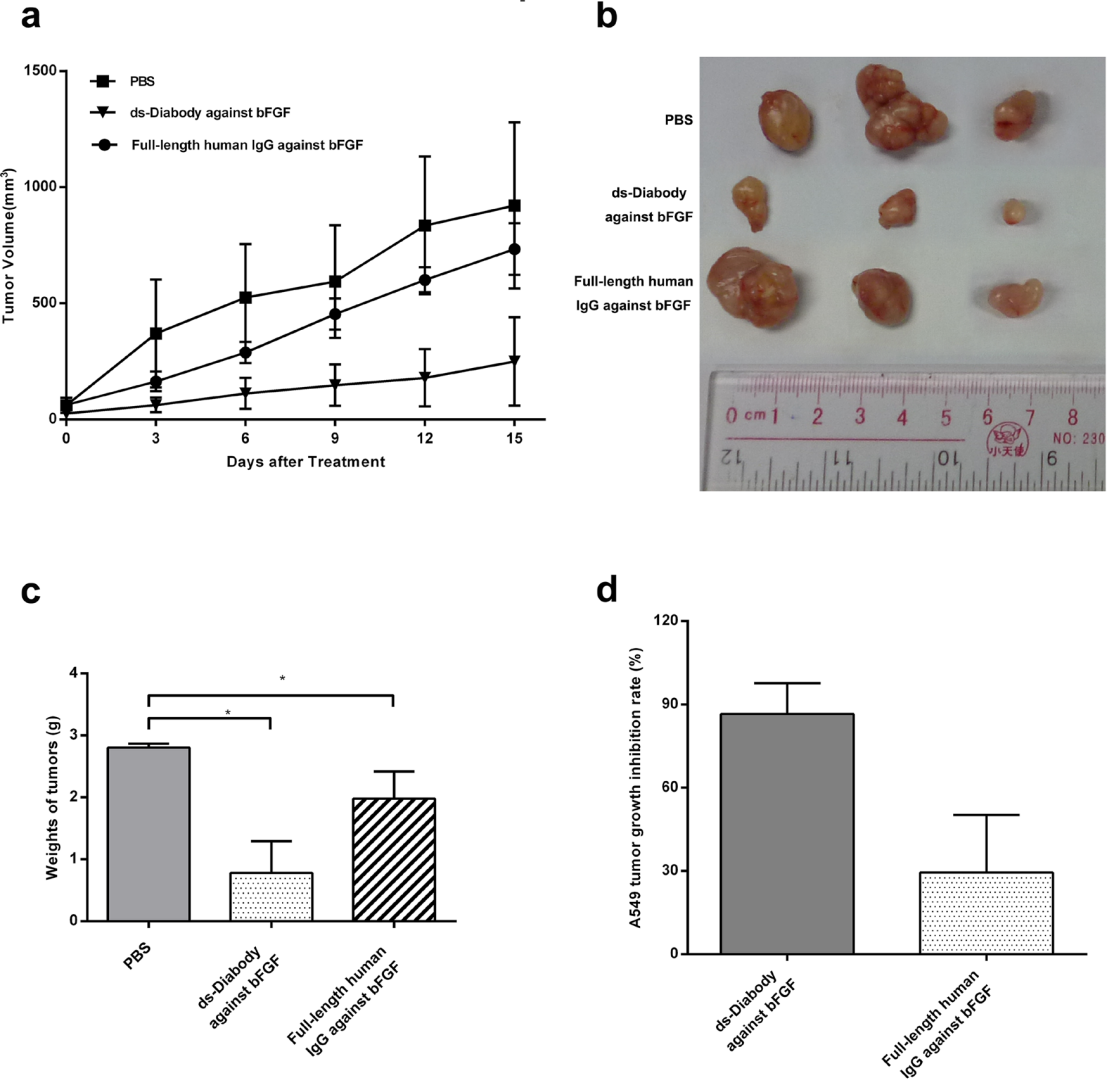
The inhibition of tumor growth by the ds-Diabody against bFGF in mice model The lung cancer cells (A549, 1×106 cells) were injected in the shoulders of BALB/c nude mice (n=6). After the tumor palpable, the mice were intravenously injected with the ds-Diabody against bFGF (10 mg/kg) six times with 3 days interval and the tumor volume was measured at different time-points after treatment. **a.** Tumor growth curve in different groups. **b.** Stripped tumors in different groups. **c.** Quantitative analysis of the tumor weight. **d.** The growth inhibition rate of tumors in different groups. The data were represented as the means ± SD (error bars) from 6 animals. *P<0.05; **P<0.01

In order to investigate the inhibitive effect of the ds-Diabody against bFGF on A549 cells growth *in vivo*, a xenograft tumor model of lung cancer was established in BALB/c nude mice. The results indicated that the administration of the ds-Diabody against bFGF could significantly inhibit the tumor growth in the tumor-bearing mice (Figure 8a-8c). Compared with the negative control group, the inhibition rate of tumor growth in the group of ds-Diabody against bFGF could reach 86.54%, while the group of the full-length human IgG against bFGF was about 29.46% (Figure 8d).

### Inhibition of tumor angiogenesis and lymphangiogenesis *in vivo* by the ds-Diabody against bFGF

**Figure 9:**
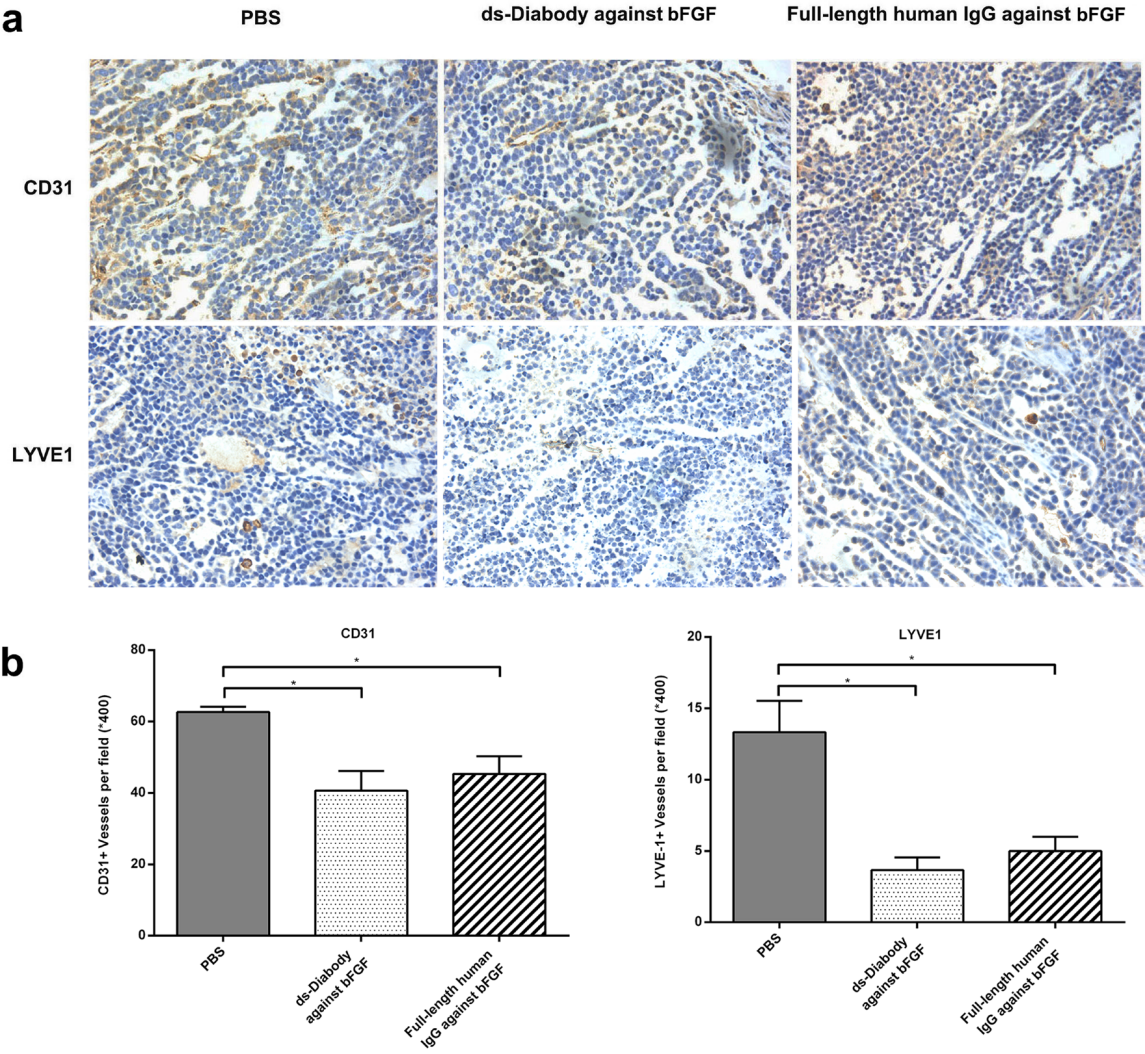
Immunohistochemical analyses of tumor tissues **a.** Paraffin sections of A549 tumors were stained for vascular endothelial cells with anti-CD31 antibody and lymphatic endothelial cells with anti-LYVE1 antibody respectively. **b.** Quantitative analysis of microvessel density and lymphatic vessels density. The number of blood vessels and lymphatic vessels at 5 high-power fields (x400) per section were counted. The data were represented as the means ± SD (error bars). *P<0.05; **P<0.01

The tumor angiogenesis and lymphangiogenesis were assessed by immunohistochemical staining with anti-CD31 and anti-LYVE1 antibodies. The average number of microvessels was about 41.0 in the group of the ds-Diabody against bFGF and 63.0 in the group of PBS. The average number of lymphatic vessels was about 4.0 in the group of the ds-Diabody against bFGF and 13.0 in the group of PBS (Figure 9). The results demonstrated that the ds-Diabody against bFGF could significantly suppress angiogenesis and lymphangiogenesis in tumor tissue.

## DISCUSSION

In our previous study, high affinity human antibodies of scFv against bFGF were selected from a phage display library, and the full-length human antibody against bFGF was reconstructed [16]. The full-length human antibody against bFGF could significantly inhibit the growth of melanoma [16]. Diabody is one of the smallest of bivalent antibody with approximately one-third the molecular weight of full-length antibody molecules, which may improve its penetration into tumor tissue [17]. In order to increase the stability of the Diabody, we constructed a human disulfide-stabilized Diabody against bFGF by introducing the disulphide bonds between the VH and VL domain by site directed mutation. The result of indirect ELISA indicated that the introducing of disulphide bonds in the variable region of diabody did not affect its antigen binding activity.

In this study, the ds-Diabody against bFGF could significantly suppress the tumor growth in nude mice and the inhibition rate could reach about 86.54%. Compared with the full-length human antibody against bFGF, the ds-Diabody against bFGF showed a stronger inhibitory effect on the growth of tumor, possibly because the ds-Diabody agasint bFGF has a stronger penetration with low molecular weight.

The signaling pathways of MAPK/ERK and PI3K/AKT are the main pathways that involved in the proliferation, migration and invasion of tumor cell [24]. Blockade of MAPK/ERK signaling pathway by anti-bFGF antibody could result in suppressing of invasion and migration triggered by bFGF [25]. We found that the ds-Diabody against bFGF could effectively inhibit the proliferation, migration and invasion of the A549 cells by blocking the signal pathway of Akt and MAPK.

In this study, we have preliminarily explored the inhibitory effect of the human ds-diabody against bFGF on the growth of A549 cells *in vitro* and *in vivo*. The results indicated that the ds-Diabody could effectively neutralize the paracrine and autocrine bFGF in tumors and block the signaling pathways of MAPK/ERK and PI3K/AKT and inhibit the tumor angiogenesis and lymphangiogenesis.

## MATERIALS AND METHODS

### Cells and animals

Human lung cancer cells (A549) were cultured in Dulbecco’s modified Eagle’s medium (DMEM) plus 10% FBS and 1% penicillin/streptomycin. All the cells were cultured in an incubator with 95% humidity and 5% CO_2_ at 37°C.

BALB/c nude mice (female, 6–8 weeks) were purchased from Laboratory Animal Center of Sun Yat-Sen University, Guangzhou, China. All the animals used in the experiments were treated humanely in accordance with Institutional Animal Care and Use Committee guidelines in Jinan University.

### Expression and purification of the ds-Diabody against bFGF

In our previous study, high affinity human antibodies of scFv against bFGF were selected from a phage display library [16]. The ds-Diabody against bFGF was constructed by site-directed mutation and overlap extension (SOE-PCR) at the position of VH44 and VL100 in the scFv. Tao has prepared a full-length human antibody against bFGF which was used as a positive control in our study [16].

The recombinant plasmid pPICZαA-ds-Diabody was transformed into *Pichia pasporis* strain GS115. The ds-Diabody against bFGF was obtained by the induction culture of the recombinant yeast transformants. The ds-Diabody against bFGF was purified from the expression supernatant by affinity chromatography and anion-exchange chromatography [26]. The target protein was assayed by SDS-PAGE and western-blot.

### Antigen binding activity of the ds-Diabody against bFGF

The 96-well plates were coated with bFGF (50 ng/well, R&D Systems) at 4°C overnight and blocked with 5% non-fat milk. The purified ds-Diabody against bFGF was diluted serially and added into the 96-well plates and incubated for 1 h at 37°C. The plates were washed 3 times with PBST (25 mM sodium phosphate, pH 7.4, 150 mM NaCl and 0.05% Tween-20) and the anti-His tag monoclonal antibody (1: 5000 dilution) was added and incubated for 1 h at 37°C. After washing, HRP-conjugated goat anti-mouse IgG was added and incubated for 30 min at 37°C. The plates were then stained with DAB (Sigma) and the absorbance values at 450 nm (A_450_) values were immediately measured in an ELISA reader.

### Cell proliferation assay

A549 cells were transferred in 96-well plates at a density of 2000 cells/well and incubated overnight at 37°C in a 5% CO_2_ incubator. After starved cultivation in DMEM with 0.5% FBS for 12 h, cells were treated with serially diluted ds-Diabody against bFGF (6.25-100 μg/mL), together with 15 ng/mL bFGF for 48 h. The control groups were treated with the full-length human IgG against bFGF or the irrelevant IgG. The number of viable cells was finally determined by Cell Counting kit-8 (CCK-8) reagent. According to the manufacturer’s protocol, 10 μL CCK-8 was quickly added to each well and incubated with cells for 2 h. After incubation, the absorbance at 450 nm was immediately measured in an ELISA reader. The cell proliferation inhibition rate was calculated.

### Western blot assay

The A549 cells (2×10^5^ cells/well) were transferred in 6-well plates and serum-starved cultured in DMEM with 0.5 % FBS overnight. The cells were exchanged with the medium of DMEM with 15 ng/mL bFGF and 0.5 % FBS and serially concentrations of the ds-Diabody and incubated for 30 min. The cells were washed with cold PBS and lysed in RIPA buffer. The lysates were centrifuged at 12000 g for 6 min at 4°C and the total proteins in the supernatant were quantified by BCA Protein Assay Kit (Thermo). The proteins were separated by SDS-PAGE and transferred to PVDF membrane (Millipore). The membrane was blocked with 5% nonfat milk at 37°C for 1 h. After washing 3 times with PBST, phosphorylation of MAPK and Akt were detected by rabbit anti p-MAPK (cell signaling #4370) and rabbit anti p-Akt (cell signaling #4060) antibodies, respectively. Total MAPK and Akt were detected by rabbit anti-MAPK (cell signaling #4695) and rabbit anti-Akt (cell signaling #4691) antibodies, respectively. The membrane was incubated with the primary antibody at 4°C overnight. After washing, the membrane was then incubated with the HRP-conjugated goat anti-rabbit IgG for 1h at 37°C. The blots were then detected with an ECL detection kit (Millipore) according to the manufacturer’s protocol. The anti-β-actin antibody (cell signaling, #4970) was used as the reference control.

### Scratch assay

The effect of the ds-Diabody against bFGF on migration of A549 cells was evaluated by scratch assay. A549 cells (5×10^5^ cells/well) suspended in DMEM complete medium were transferred in 6-well plates. After incubated for 24 h, a scratch treatment was taken. After washing with serum-free DMEM, the DMEM with 0.5% FBS and ds-Diabody against bFGF (100 μg/mL) was added to the wells. The controls were the full-length human IgG against bFGF (100 μg/mL) and the irrelevant IgG (100 μg/mL) and the medium only. After incubated for 0, 12 and 24 h, the plates were imaged with a computerized imaging system. The cell migration rate of each group was calculated.

### Invasion assay

The effect of the ds-Diabody against bFGF on invasion of A549 cells was assayed in transwell chambers. The upper side of the filters was coated with 45 μl matrigel matrix (BD) diluted (1:3) with serum-free DMEM. After serum-starved for 12 h, A549 cells were seeded (5×10^4^ cells/well) onto the layer of Matrigel using serum-free medium, then stimulated with 15 ng/mL bFGF and 100 μg/mL ds-Diabody against bFGF for 16 h. The control groups were the full-length human IgG against bFGF (100 μg/mL), the irrelevant IgG (100 μg/mL) and the medium only. The lower chambers were DMEM with 10% FBS. At the end of the treatment, cells on the upper side of the filters were mechanically removed, and those migrated onto the lower side were fixed with 70% ethanol, stained by 0.1% crystal violet (Meryer) and imaged with a computerized imaging system.

### Tumor model

Human lung cancer A549 cells (1×10^6^ cells in 100 μl) were subcutaneously injected into the right shoulder flank of BALB/c nude mice (15-20 g, n = 6). When palpable tumors (≥5 mm in diameter) developed, the tumor-bearing mice were intravenously injected with 10 mg/kg ds-Diabody against bFGF or the full-length human antibody against bFGF or PBS six times at 3 days intervals. Tumor size was measured every 3 days in two dimensions using a vernier caliper. Mice were euthanized 24 hours later after the last administration. The tumors were stripped for immunohistochemistry and weighed to calculate the tumor growth inhibition rate. Tumor volume (mm^3^) was calculated as V = 1/2(a × b^2^). a = tumor length, b = tumor width; Tumor growth inhibition rate =(1 - the average tumor weight of treated groups/the average tumor weight of PBS group)× 100%.

### Evaluation of microvessel density and lymphatic vessels density in subcutaneous tumors

Tumor tissues were fixed in 4% paraformaldehyde, embedded in paraffin, and cut at 5 μm. After deparaffinization, the sections were heat for 10 min at 100°C in sodium citrate buffer (10 mM, pH 6.0) for antigen retrieval and treated with 3% H_2_O_2_ for 15 min at room temperature to block endogenous peroxidase activity. The sections were blocked in 3% bovine serum albumin (BSA) for 1 h and incubated with primary antibodies overnight at 4°C (rabbit anti-CD31 polyclonal antibodies and rabbit Anti-LYVE1 polyclonal antibody, Abcam). The sections were then incubated with secondary antibody of biotinylated goat anti-rabbit polyclonal antibody and avidin-HRP for 1 h. The sections were stained with 3, 3′-diaminobenzidine (DAB) and hematoxylin. The vessels density was determined by counting the number of microvessels and lymphatic vessels in five random high-power fields within the sections, as described [12].

### Statistics analysis

Statistical comparisons were analyzed by one-way analysis of variance (ANOVA) followed by the least significant difference (LSD) test. The data were represented as mean ± SD. *P*-values < 0.05 (*) and *P*< 0.01 (**) were considered statistically significant.
